# Simultaneous analysis of five triterpenes in *Centella asiatica* by high performance liquid chromatography with cyclodextrins as the mobile phase additives

**DOI:** 10.1038/s41598-020-75554-z

**Published:** 2020-10-29

**Authors:** Changhe Wang, Yanan Zhao, Ruomeng Yang, Haijing Liu

**Affiliations:** 1 Shaanxi Institute for Food and Drug Control, No 21, Keji Fifth Road, Xi’an, 710065 Shaanxi Province China; 2 Nanjing Aureole Pharmaceutical Co., Ltd., No.11, Changchun Road, High Tech Zone, Zhengzhou, 450001 Henan Province China; 3 Xi’an University, No 1, Keji Sixth Road, Xi’an, 710065 Shaanxi Province China

**Keywords:** Drug discovery, Chemistry

## Abstract

Triterpenes are considered the major active components in *Centella asiatica* (L.) Urb. (*C. asiatica*), such as asiatic acid, madecassic acid, asiaticoside, madecassoside and asiaticoside B. It is difficult to simultaneously determine five triterpenes because of madecassoside isomers (madecassoside and asiaticoside B), and the great polarity difference between triterpene acid and triterpene glycoside. In this study, a simple high performance liquid chromatography method with isocratic elution employing cyclodextrins (CDs) as the mobile phase additives was developed to determine five triterpenes in *C. asiatica*. Various factors affecting triterpenes retention in the C18 column, such as the nature of CDs, γ-CD concentration, acetonitrile percentage and temperature, were studied. Experimental results showed that γ-CD, as an effective mobile phase additive, could markedly reduce the retention of triterpenes (especially asiatic acid and madecassic acid), and improve the separation for madecassoside and asiaticoside B. The elution of five triterpenes could be achieved on an ODS C18 column within 30 min using the acetonitrile-0.2% phosphoric acid contained 4.0 mM γ-CD (20:80, v/v) mixture as the mobile phase. The retention modification of triterpenes may be attributed to the formation of the triterpenes-γ-CD inclusion complexes. The optimized method was successfully applied for simultaneous determination of five triterpenes in *C. asiatica*.

## Introduction

*Centella asiatica* (L.) Urb. (*C. asiatica*) is an ethnomedicinal herbaceous species that grows abundantly in China, India, Southeastern Asian and Africa. In China, it has been a long history of therapeutic uses, e.g., dampness-heat jaundice, heat stroke diarrhea, wound healing^1–4^. In recent years, its extracts and bioactive components have been reported to have anti-inflammatory^5^, anti-tumor^6,7^, anti-oxidant^8^, wound healing^9^, cardioprotective^10^ and improving-memory effect^11,12^. Triterpenes are considered to be the major active components in *C. asiatica*, such as asiatic acid, madecassic acid, asiaticoside, madecassoside and asiaticoside B^13,14^. Asiaticoside and madecassoside have been the marker compounds of *C. asiatica* in Chinese Pharmacopoeia (2015 edition). However, the content variability of triterpene acids and glycosides has been very obvious in different samples of *C. asiatica* from various geographical regions and cultivation conditions because of biotransformation of triterpenes^15^. So, the quantification analysis of five prominent triterpenes in *C. asiatica* is very important.

**Figure 1:**
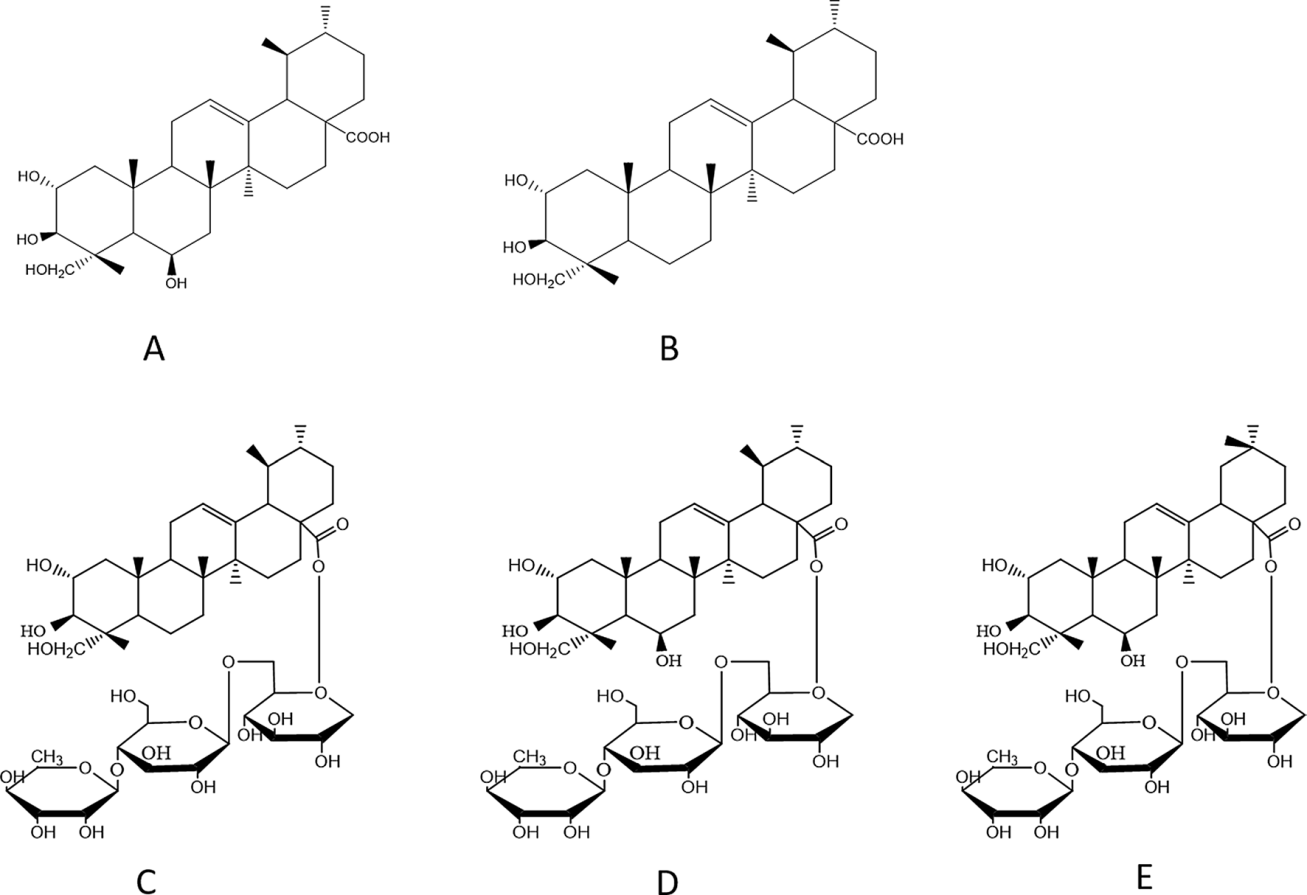
Chemical structures of madecassic acid (**A**), asiatic acid (**B**), asiaticoside (**C**), madecassoside (**D**), asiaticoside B (**E**).

Most studies for quantitative analysis of triterpenes in *C. asiatica* are commonly carried out by high-performance liquid chromatography (HPLC). However, these methods could only determine two, three or four constituents^16–19^. Lu et al. proposed a HPLC method with isocratic elution only dedicated for the quantification of madecassoside and asiaticoside^16^. An addition of β-cyclodextrin in the mobile phase was used for the quantification of madecassoside and asiaticoside B^17^. HPLC methods with gradient elution were developed by Shen et al. and Rafamantanana et al. for simultaneous quantification of three triterpenes (asiaticoside, madecassoside and asiatic acid) and four triterpenes (asiaticoside, madecassoside, asiatic acid and madecassic acid), respectively^18,19^. But, there is no report on the simultaneous quantification of five triterpenes in *C. asiatica* using HPLC. Compared with the glycosides, the hydrophobic character of triterpene acids is strong and it results in robust retention on a nonpolar stationary phase and a longer retention time for triterpene acids. So, the simultaneous quantification of triterpenoid glycosides and aglycones is often carried out by HPLC methods with gradient elution. In addition, madecassoside and asiaticoside B are the isomeric compounds (Fig. 1). Therefore, it is very difficult to develop a HPLC method with isocratic elution for simultaneous quantification of five triterpenes in *C. asiatica.*

Cyclodextrins (CDs) are cyclic oligosaccharides composed of six or more glucopyranosyl units through α-1,4-glycosidic bonds. They present a hollow truncated cone, which the large and small openings are exposed to the secondary and primary hydroxyl groups, respectively. The outside of the cavity is hydrophilic, whereas the inner is hydrophobic. So, CDs are able to host hydrophobic compounds, and increase water solubility of them or alter other properties. As the mobile phase additives in HPLC, CDs can form the inclusion complexes with analytes, change the retention behavior of analytes, and be available for separation enantiomers and geometric isomers^20–22^ or simultaneous analysis of glycosides and aglycone with isocratic elution^23,24^. Therefore, the addition of CDs into the mobile phase was investigated to alter the retention behavior of five triterpenes allowing simultaneous analysis of five triterpenes in *C. asiatica* using HPLC with isocratic elution.

Bearing in mind the above, the main objective of this study is to develop a HPLC method with isocratic elution employing CDs as the mobile phase additives for simultaneous determination of five triterpenes (asiatic acid, madecassic acid, asiaticoside, madecassoside and asiaticoside B) in *C. asiatica*. Various factors affecting the retention of five triterpenes in the C18 column, such as the nature of CDs, CDs concentration, acetonitrile percentage and temperature, were also studied.

## Materials and methods

### Materials

Asiaticoside and madecassoside were obtained from China's National Institute for Food and Drug Control (Beijing, China). Asiatic acid, madecassic acid and asiaticoside B were obtained from Xi'an Kailai Bioengineering Co., Ltd. (Xi’an, China). α-CD, β-CD, hydroxypropyl-β-CD (HP-β-CD), glucosyl-β-CD (Glu-β-CD) and γ-CD were purchased from JINGYE Biotech & Pharmaceutical Co., Ltd. (Xi’an, China). HPLC-grade acetonitrile was purchased from Tianjin Kermel Chemical Reagent Co., Ltd. (Tianjin, China). High purity water was purified using a Milli-Q50 SP Reagent Water System (Millipore Corporation, Billerica, MA, USA). All other chemicals were of analytical reagent grade, from Xi’an Analytical Instrument Factory (Xi’an, China). *C. asiatica* was purchased from the different TCM Store located at Xi’an (China) and stored at room temperature before use. The authenticity of the plant species of these herbs was authenticated by professor Zengjun Guo (Shool of Pharmacy, Xi’an Jiaotong University).

### Preparation of standard substance solution

The stock solution of each analyte was prepared by dissolving asiatic acid for 2.0 mg, 4.0 mg for madecassic acid, 10.0 mg for asiaticoside, 10.0 mg for madecassoside and 10.0 mg for asiaticoside B in 20 mL methanol, five solutions were stored at − 20 °C and found to be stable for at least 3 months. The standard solution (20 µg/mL for asiatic acid, 40 µg/mL for madecassic acid, 100 µg/mL for asiaticoside, 100 µg/mL for madecassoside and 100 µg/mL for asiaticoside B) was prepared daily by dilution of the stock solution with methanol and stored at − 4 °C during the day.

### Preparation of samples

The *C. asiatica* was dried in an oven at 60 °C for 24 h. Dried materials were powdered by a disintegrator (HX-200A, Yongkang Hardware and Medical Instrument Plant, China) and then sieved (40–60 mesh). The powders of *C*. *asiatica* (0.2 g) were accurately measured and extracted in a Soxhlet extractor with 100 ml petroleum ether for 4 h to remove chlorophyll, and the petroleum ether solution was discarded. Subsequently, the powders were refluxed with 100 ml ethanol for 6 h. The extract was evaporated to dryness under vacuum at 40 °C, and re-dissolved in 25 mL water. The obtained aqueous solution was extracted three times by 25 ml n-butanol saturated with water. The n-butanol solutions were combined and evaporated to dryness on water bath, and the residue was re-dissolved in methanol. After filtration through a 0.22 µm Millipore membrane filter, 10 µL methanol solutions were injected into HPLC.

### Equipment and chromatographic conditions

HPLC analysis was performed using a Shimadzu 20A series (Shimadzu, Kyoto, Japan), which is consisted of a LC-20AT pump, a SPD-20A UV–VIS detector, a model 7725i Rheodyne sample injection valve (Cotati, CA, USA) fitted with a 20 μL injection loop, and a CBM-20A system controller. The chromatographic column was an Inertsil ODS C18 column (250 mm × 4.6 mm id, 5 μm) provided by GL Sciences Inc. (Japan). The temperature of the column was controlled with an AT-330 column heater (Tianjin Autoscience Instrument Co. Ltd., Tianjin, China). Data was analyzed using LC solution 15C (Shimadzu, Kyoto, Japan).

The mobile phases consisted of acetonitrile and 0.2% phosphoric acid solution containing 0.0, 1.0, 2.0, 4.0 or 6.0 mM CDs, were prepared by dissolving known amounts of CDs (α-CD, β-CD, HP-β-CD, Glu-β-CD or γ-CD) in 0.2% phosphoric acid solution and mixing with acetonitrile. The pH of the mobile phase is about 1.90. Before being used, the mobile phases were filtered through a polypropylene membrane with 0.22 μm (Whatman, Maidstone, UK) and sonicated for approximately 15 min. The flow-rate was 1 mL·min^−1^. The detection wavelength was 205 nm.

Influence of various factors on the retention of five triterpenes in the C18 column, such as the nature of CDs, CDs concentration, acetonitrile percentage and temperature, were evaluated by capacity factors (*k*) and resolution (*R*). The retention factor can be calculated according to the following formula:

$$k = \frac{{t_{R} - t_{0} }}{{t_{0} }}$$

where *k* is retention factor of analyte, *t*_*R*_ is the retention time of analyte, and *t*_*0*_ is the void time, respectively. The *R* value of two adjacent peaks is not less than 1.5, which means that the two peaks are completely separated.

### Method validation

Standard solutions of five triterpenes were prepared by dissolving an appropriate amount of each triterpene in methanol and diluted to appropriate concentrations with methanol. Linearity within a range of six concentrations of five triterpenes was verified by five replicate injections. The limits of detection (LOD) and quantification (LOQ) were estimated for each triterpene at signal-to-noise ratio (S/N) of 3 and 10, respectively. The intra-day and inter-day precisions of five triterpenes were investigated by five replicated injections on day 1 and consecutive day 5, respectively. The accuracy of the method was assessed by the recovery test. The powders of *C*. *asiatica* (0.1 g) spiked with the accurate amounts of five triterpenes were extracted and analyzed as described in "Preparation of samples" and "Equipment and chromatographic conditions sections". The spiked amounts of five triterpenes were similar to their concentration in the sample. The recoveries for five triterpenes were calculated as follows: recovery (%) = 100 × (amount found—original amount)/amount spiked.

## Results and discussion

### Influence of CD nature on the retention

**Figure 2:**
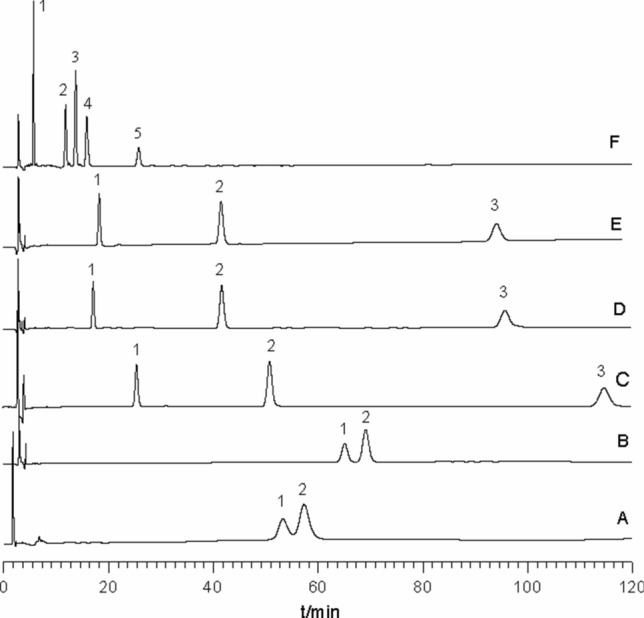
HPLC chromatograms of a standard mixture of five triterpenes. Column: ODS C18 column (250 × 4.6 mm id, 5 μm). Mobile phase: acetonitrile—0.2% phosphoric acid contained 4 mmol/L cyclodextrins (20:80, v/v), cyclodextrins: A, no CDs; B, α-CD; C, HP-β-CD; D, β-CD; E, Glu-β-CD; F, γ-CD. Flow-rate: 1 mL/min. Temperature: 25 °C. Wavelength of detection: 205 nm. Analytes: 1, asiaticoside B; 2, madecassoside; 3, asiaticoside; 4, madecassic acid; 5, asiatic acid.

The retention behaviors of asiatic acid, madecassic acid, asiaticoside, madecassoside and asiaticoside B in C18 column were investigated using the acetonitrile-0.2% phosphoric acid (20:80, v/v) mixture as the mobile phase and CDs (α-CD, β-CD, Glu-β-CD, HP-β-CD and γ-CD) as additives. Asiaticoside B and madecassoside were eluted in 60 min, but other triterpenes were strongly retained on the chromatographic column and were not eluted in 120 min using the mobile phase contained no CDs (Fig. 2A). Asiaticoside B and madecassoside were eluted in 120 min using α-CD as the mobile phase additive (Fig. 2B), and three triterpenes (asiaticoside B, madecassoside and asiaticoside) were eluted in 120 min using β-CD, HP-β-CD or Glu-β-CD used as the mobile phase additives (Fig. 2C, D, E). Fortunately, five triterpenes were all eluted in 30 min using γ-CD as the mobile phase additive (Fig. 2F), and the order of the elution was asiaticoside B (5.80 min), madecassoside (11.92 min), asiaticoside (13.83 min), madecassic acid (15.94 min) and asiatic acid (25.85 min).

Asiaticoside B and madecassoside are a pair of isomeric compounds. From the previous studies, the isomers can only become single peak in common HPLC because of their similar characters^19^. In this study, the baseline separation of asiaticoside and asiaticoside B could not be achieved in 60 min without CDs additives. When α-CD, β-CD, HP-β-CD or Glu-β-CD was as a mobile phase additive, the complete separation of asiaticoside B and madecassoside was obtained, and the resolution were shown in Table 1. In addition, β-CD, HP-β-CD and Glu-β-CD could decrease their retention time. These results are similar to the results of Pan et al^17^. But, the above four CDs could not change the retentions of madecassic acid and asiatic acid. Compare to γ-CD, triterpenes do not fit into the smaller cavity of α-CD (or β-CD), and the inclusion complexes do not form. So, madecassic acid and asiatic acid was not eluted with α-CD, β-CD, HP-β-CD or Glu-β-CD as a mobile phase additive. The improvment of separation and the decrease of the retention time for asiaticoside B and madecassoside may be attributed to that CDs (β-CD, HP-β-CD and Glu-β-CD) can form hydrogen bond with glucose in asiaticoside B and madecassoside. γ-CD not only improved the separation of asiaticoside B and madecassoside, but also decreased sharply the retention time of five triterpenes. This phenomenon can be interpreted by the formation of the inclusion complexes between γ-CD and five triterpenes. Thus, the resolution of triterpene isomers may be improved by a second mechanism based on the differences in hydrophobicity between analytes. On the other hand, the complexation phenomenon increases the hydrophilicity of them in the mobile phase and then significantly decreases their retention time in the column. This is consistent with our previous report that γ-CD can form the inclusion complexes with triterpenes (oleanolic acid and ursolic acid), and decrease sharply their retention time and improve their separation^22^.

**Table 1:** The resolution (*R*) of madecassoside and asiaticoside B in HPLC imploying cyclodextrins (CDs) as the mobile phase additives.

	α-CD	β-CD	HP-β-CD	Glu-β-CD	γ-CD
*R*	1.89	21.90	18.53	22.88	14.18

### Influence of γ-CD concentration on the retention

**Figure 3:**
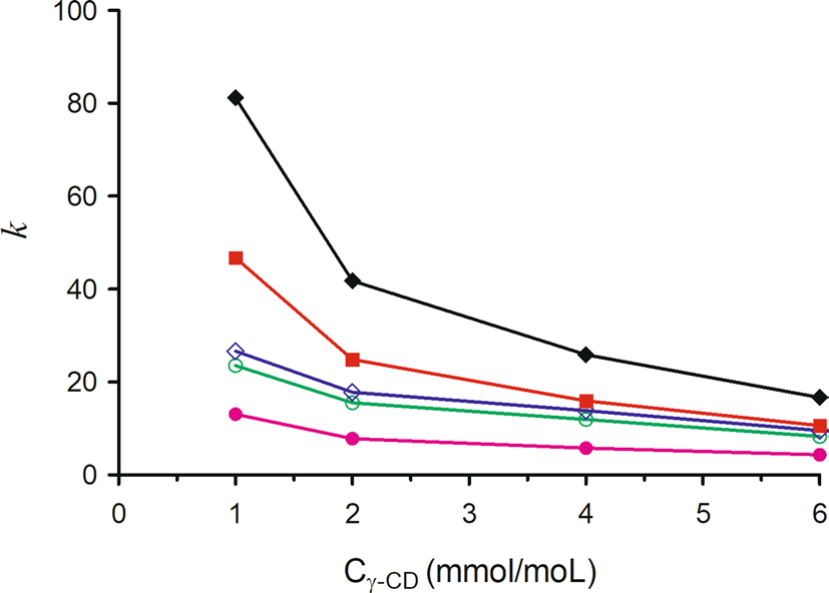
Effect of γ-CD concentrations on the retention factors (*k*) of five triterpenes in HPLC. Mobile phase: acetonitrile—0.2% phosphoric acid contained γ-CD (20:80, v/v), γ-CD concentration: 1, 2, 4, 6 mM. Other conditions as in Fig. 2. Analytes: (filled diamond) asiatic acid, (filled square) madecassic, (diamond) asiaticoside, (circle) madecassoside, (filled circle) acidasiaticoside B.

The concentration of γ-CD concentration is a governing factor in the elution of analytes because it determined the extent of formation of the inclusion complexes. To verify the effect of γ-CD on the retention behavior of five triterpenes, the experiments were performed with the acetonitrile-0.2% phosphoric acid contained the different concentration of γ-CD (20:80, v/v) mixture as the mobile phase, the capacity factors (*k*) of five triterpenes were calculated and plots of *k* versus the concentrations of γ-CD were constructed. Figure 3 shows that the *k* values of five triterpenes decreased significantly with the increase of γ-CD concentrations from 1.0 to 6.0 mM, and the decreased magnitudes of triterpene acids are more than those of triterpene glycosides. The molecules of triterpene glycoside are bulky and the hydrophobicity is weak due to the contribution of a glucoside moiety, so the triterpene acid/γ-CD complexes are more stable than the triterpene glycoside/γ-CD complexes, and the decrease magnitude of triterpene acid in the retention are more than those of triterpene glycosides. Five triterpenes were all eluted in 30 min, and the separation for five triterpenes was satisfactory when the γ-CD concentration was 4.0 mM. When the γ-CD concentration was more than 4 mM, the retention time was too short and the separation for three triterpene glycosides was not satisfactory. Asiaticoside B and madecassoside were completely separated with γ-CD concentrations from 1.0 to 4.0 mM, and the R values of asiaticoside B and madecassoside were all higher than 11. Therefore, the γ-CD concentration in the mobile phase was chosen as 4.0 mM.

### Influence of the volume fractions of acetonitrile on the retention

**Figure 4:**
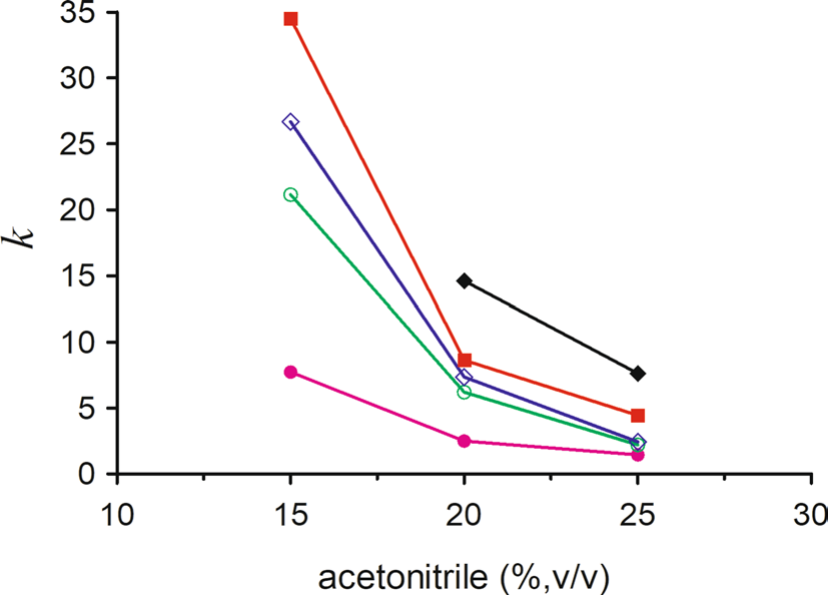
Effect of acetonitrile volume fractions in the mobile phase on the retention factors (*k*) of five triterpenes in HPLC. Mobile phase: acetonitrile—0.2% phosphoric acid contained 4 mmol/L γ-CD, acetonitrile volume fractions (v/v): 15, 20, 25%. Other conditions as in Fig. 2. Analytes: (filled diamond) asiatic acid, (filled square) madecassic, (diamond) asiaticoside, (circle) madecassoside, (filled circle) acidasiaticoside B.

The proportion of organic solvent in the mobile phase could significantly influence the retention of analytes. In order to investigate the effect of acetonitrile in the mobile phase on the retention behavior of five triterpenes, 15–25% (v/v) acetonitrile was chosen. Figure 4 shows the *k* values of five triterpenes obtained using the mobile phases prepared by mixing the appropriate amount of acetonitrile and 4 mM of γ-CD solutions. When the volume fraction of acetonitrile was 15%, asiatic acid was not eluted in 120 min. The *k* values of five triterpenes sharply decreased when the volume fraction of acetonitrile increased, and it suggested that the retention time for five triterpenes decreased with the increase of acetonitrile concentration. When the volume fraction of acetonitrile was to 25%, the retention times of triterpene glycosides were too short, and the separation for madecassoside and asiaticoside was not satisfactory and the R value was lower than 1.5. Therefore, the most suitable volume fraction of acetonitrile in the mobile phase was 20% (v/v).

### Effect of temperature on the retention

**Figure 5:**
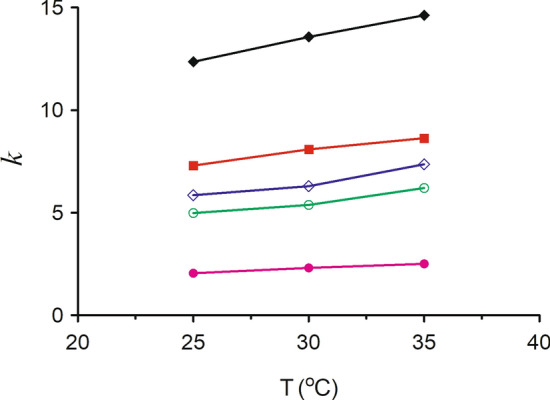
Effect of temperature on the retention factors (*k*) of five triterpenes in HPLC. Mobile phase: acetonitrile—0.2% phosphoric acid contained 4 mmol/L γ-CD (20:80, v/v). Temperature: 25, 30, 35. Other conditions as in Fig. 2. Analytes: (filled diamond) asiatic acid, (filled square) madecassic acid, (diamond) asiaticoside, (circle) madecassoside, (filled circle) asiaticoside B.

Temperature could also affect the retention and separation of analytes. To verify the effect of temperature on the retention behavior of five triterpenes, the experiments were performed with the acetonitrile-0.2% phosphoric acid contained 4.0 mM γ-CD (20:80, v/v) mixture as the mobile phase at the different temperature (25 °C, 30 °C and 35 °C). Figure 5 shows the *k* values of five triterpenes at the different temperature. The *k* values of five triterpenes increased with the increase of temperature, and it suggested that the retention time for five triterpenes increased and the eluting ability of the mobile phase was weaker with the increase of temperature. It may be attributed to that the inclusion of CDs and triterpenes is an exothermic process, and the formation of the inclusion complexes between γ-CD and triterpenes is unfavor to higher temperature. Asiaticoside B and madecassoside were completely separated with temperature from 25 to 35 °C, and the R values of asiaticoside B and madecassoside were all higher than 13. So, the temperature was settled at 30 °C.

### Method validation

The linear regression analysis was performed by five replicate injections at six concentration levels of five triterpenes. The calibration curves of five triterpenes, constructed by plotting the peak area of each analyte vs. analyte amount, both exhibit good linearity (*r*^2^ ≥ 0.9996) over the concentration range (shown in Table 2). The limit of detection (LOD) and the limit of quantification (LOQ) under the chromatographic analysis at signal-to-noise ratio (S/N) of 3 and 10, respectively, were determined for five triterpenes. The LOD and LOQ for the developed HPLC with isocratic elution are in the range of 0.5–3 ng and 1.5–8 ng, respectively. The intra-day and inter-day precisions of five triterpenes were examined by five replicated injections on day 1 and consecutive day 5, respectively. The intra-day and inter-day precisions of five analytes in the *C. asiatica* sample both are less than 2%.

**Table 2:** Results of HPLC method validations for five triterpenes.

Triterpenes	Linearity equation	*r*^2^	Test range (ng)	LOQ (ng)	LOD (ng)	Intraday precision (RSD, %)	Interday precision (RSD, %)	Recovery (%, n = 6)
Asiaticoside	y = 5749x + 8480	0.9996	50–5000	1.5	0.5	1.64	1.45	99.3 ± 1.01
Asiaticoside B	y = 3609x + 12,781	0.9998	50–5000	2	1	0.53	1.62	99.6 ± 1.37
Madecassoside	y = 6278x-3486	0.9998	50–5000	2	1	1.31	1.68	98.4 ± 1.25
Madecassic acid	y = 24076x + 13,033	0.9996	20–2000	5	2	1.09	1.17	98.7 ± 1.48
Asiatic acid	y = 25974x + 4978	0.9996	10 -1000	8	3	0.86	1.31	98.2 ± 1.73

Meanwhile, the recovery test was performed to examine the accuracy of the extraction method. The accurate amounts of five triterpenes were spiked to certain amounts (0.1 g) of *C. asiatica* powder, then extracted as described in the preparation of samples, and analyzed. The percent recoveries for the analytes were calculated as follows: recovery (%) = 100 × (amount found—original amount)/amount spiked. As shown in Table 2, the developed HPLC method employing γ-CD as a mobile phase additive had good accuracy within 98.2% ± 1.73% and 99.6% ± 1.37% for all five triterpenes in *C. asiatica* samples.

### Application

**Figure 6:**
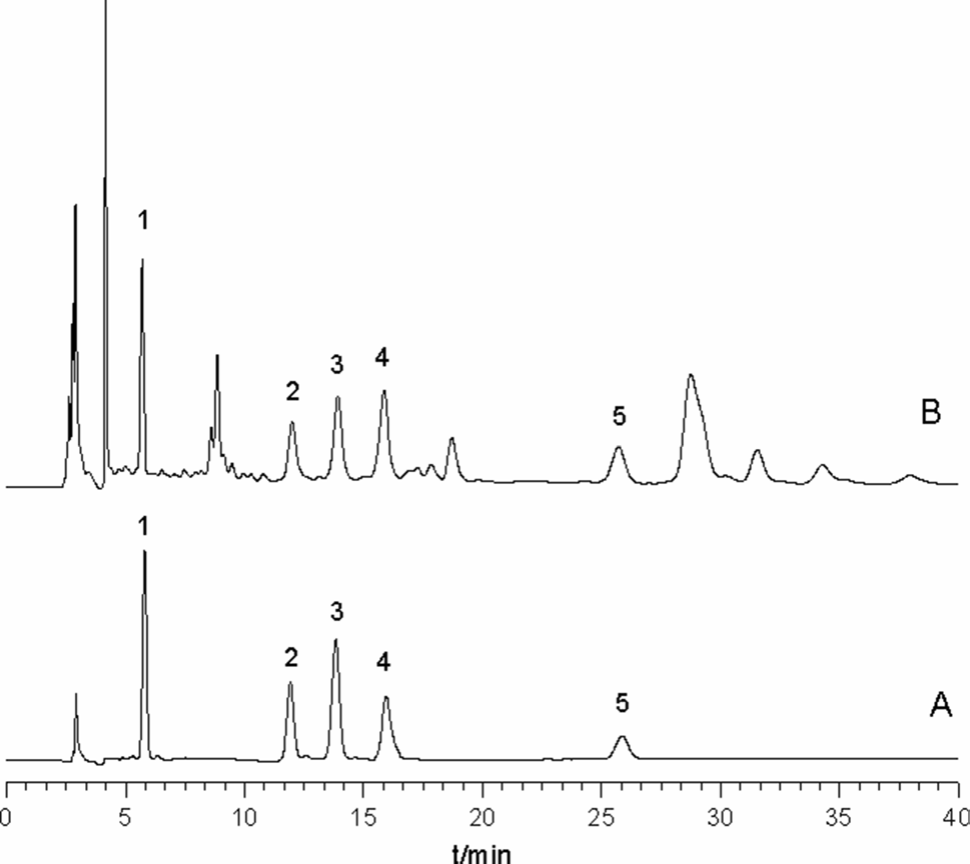
Typical HPLC chromatograms of a standard mixture of five triterpenes (**A**) and *Centella asiatica* (L.) Urb. (**B**). Analytes: 1, asiaticoside B; 2, madecassoside; 3, asiaticoside; 4, madecassic acid; 5, asiatic acid.

The developed HPLC employing γ-CD as a mobile phase additive were applied to separate and determine five triterpenes in *C. asiatica*. The typical chromatograms are shown in Fig. 6. The peaks for five triterpenes were identified by retention time and UPLC/MS. The retention time of asiaticoside B, madecassoside, asiaticoside, madecassic acid and asiatic acid were 5.80 min, 11.92 min, 13.83 min, 15.94 min and 25.85 min, respectively. Assay results are summarized in Table 3. These results show the outstanding differences in the content of five triterpenes. It may be attributed to the difference of environment and climate for habitats, harvest seasons and triterpenes biotransformation of *C. asiatica*.

**Table 3:** Contents of five triterpenes in the real samples (mg/g dry powder, n = 3).

Samples	Asiaticoside	Asiaticoside B	Madecassoside	Madecassic acid	Asiatic acid
Reference *C. asiatica*	4.88 ± 0.17	4.73 ± 0.19	5.02 ± 0.21	3.15 ± 0.14	2.64 ± 0.12
Commercial *C. asiatica *Shaanxi	5.22 ± 0.15	3.07 ± 0.11	3.53 ± 0.12	1.75 ± 0.06	1.66 ± 0.07
Commercial *C. asiatica *Jiangsu	4.51 ± 0.11	4.60 ± 0.15	4.82 ± 0.15	3.07 ± 0.16	2.35 ± 0.08
Commercial *C. asiatica *Yunnan	4.84 ± 0.13	3.49 ± 0.12	2.75 ± 0.11	3.67 ± 0.09	3.53 ± 0.11

The developed method was successfully applied for simultaneous determination of five triterpenes in *C. asiatica*. But, the developed method has the limitations as follows. High concentration CDs have great damage to chromatographic column. This method is not suitable for mobile phases with a high proportion of organic phases.

## Conclusions

It is very difficult to simultaneously analyze five triterpenes in *C. asiatica* using HPLC with isocratic elution because of madecassoside isomers (madecassoside and asiaticoside B), and the great polarity difference between triterpene acid and triterpene glycoside. CDs can form the inclusion complexes with analytes, and increase water solubility and change retention behavior of analytes. In this work, we propose a HPLC method with isocratic elution for simultaneous quantification of five triterpenes employing CDs as the mobile phase additives. Our results show that as a very effective mobile phase additive, γ-CD can markedly reduce the retention of triterpenes (especially madecassic acid and asiatic acid), and improve the separation for asiaticoside B and madecassoside. The retention behavior of triterpenes not only depends on the nature concentration of CDs, but also the property of triterpenes and temperature. The proposed HPLC method with isocratic elution was validated. Five triterpenes exhibit good linearity (*r*^2^ ≥ 0.9996) over the concentration range, and the intra-day and inter-day precisions of five triterpenes both are less than 2%. The proposed HPLC method has good accuracy within 98.2% ± 1.73% and 99.6% ± 1.37% for five triterpenes in *C. asiatica* samples. The proposed method was successfully applied for simultaneous determination of five triterpenes in *C. asiatica*, and offers a good alternative for quality assessment of the herb.
